# An empirical long-term competition among natural yeast isolates reveals that short-term fitness largely but not entirely predicts long-term outcomes

**DOI:** 10.1101/2025.10.09.681448

**Published:** 2025-10-10

**Authors:** Alexandra N Khristich, Olivia M Ghosh, Jean CC Vila, Shaili Mathur, Abhishek Dutta, Marion Garin, Joseph Schacherer, Dmitri A Petrov

**Affiliations:** 1 Department of Biology, Stanford University, Stanford CA 94305, USA; 2 Department of Physics, Stanford University, Stanford CA 94305, USA; 3 Université de Strasbourg, CNRS, GMGM UMR 7156, Strasbourg, France; 4 Institut Universitaire de France (IUF), Paris, France; 5 Chan Zuckerberg Biohub, San Francisco, CA 94158, USA

## Abstract

In this study, we investigate the relative contribution of initial fitness to the long-term success of a genotype competing in a naturally diverse population. Specifically, we compete over 300 genetically barcoded *S. cerevisiae* isolates in a pooled setting for over 700 generations. We found that the strains that remain at detectable frequency until the end of the competition uniformly come from the top 95th percentile in the initial fitness values, making initial fitness the most significant predictor of long-term success. However, we occasionally see heterogeneity in the competition outcomes, which suggests a role of stochastic adaptation, clonal interference, and possibly frequency-dependent changes in strains’ fitness. We demonstrate that the “finalists” of our competition change on the genetic level, and that the spectrum of *de novo* mutations depends both on the strains’ genotype and environment. Finally, we show that gene targets of the novel mutations are specific to the combination of strain identity and environment, even among the genetically similar strains and environments that select for the same strains in the beginning of the competition.

## Introduction

Predicting the long-term outcomes of evolution is a central goal in evolutionary biology (Wortel et al., 2023). This is difficult because many different factors can influence the fate of a lineage in a population. Among these are initial fitness, ecological interactions with other lineages, and the ability to adapt in the future, or “evolvability.” The relative significance of these factors on the chances of long-term success remains poorly understood. In other words, “short-term” fitness, while important for predicting the proximate dynamics of evolution, may not be sufficient for predicting a lineage’s long-term survival.

Previously, microbial evolution experiments with clonal populations have demonstrated that in the short term, population dynamics are largely predictable at the statistical level, with the fittest strains taking over and outcompeting neighboring strains (Levy et al., 2015; Nguyen Ba et al., 2019). However, a large body of theoretical work has shown that in the long run, differences in evolvability and emergent ecological dynamics can have major implications for evolutionary outcomes (Kirschner and Gerhart, 1998; Earl and Deem, 2004; Draghi et al., 2010; Ferrare and Good, 2024). Indeed, experimental work has shown that over longer timescales, factors other than initial fitness become important. For example, the famous *E. coli* long-term evolution experiment (Wiser, Ribeck and Lenski, 2013) revealed that during evolutionary competition mutator alleles that alter the beneficial mutation rate arise (Woods et al., 2011), and nascent ecological dynamics and abundant frequency-dependence emerge (Ascensao et al., 2023, 2025). In addition, countless experiments investigating the relationship between initial fitness and the fitness effects of future mutations revealed a universal phenomenon known as diminishing returns epistasis: adaptive mutations of larger effect are more likely to arise in the strains with initially low fitness (Chou et al., 2011; Khan et al., 2011; Wiser, Ribeck and Lenski, 2013; Couce and Tenaillon, 2015; Ardell et al., 2024). Yet, the effect of diminishing returns epistasis on long-term adaptation dynamics remains unclear.

While these empirical examples lend some insight into the relationship between initial fitness and long-term success, they are all either clonal populations or progeny of single crosses evolving in isolation from each other. Hence, they interrogate populations with a constrained pool of initial diversity, both genetically and phenotypically.

Here, we investigate the role of “short-term” fitness in determining evolutionary success for a collection of natural *S. cerevisiae* isolates, spanning the full genetic and ecological range of the species. Perhaps natural strains are no different from closely related strains in their evolutionary dynamics, and the standing genetic variation in initial fitness trumps any differences in evolvability traits or the effects of frequency-dependent fitness changes. In this scenario, short-term fitness would be a perfect predictor of long term success, and the initially fittest strain will always take over the population. But perhaps existing variation in other traits across a species, such as ploidy (Zhu, Sherlock and Petrov, 2016; Peter et al., 2018; Sharp et al., 2018), mutation rate (Gou, Bloom and Kruglyak, 2019; Jiang et al., 2021), heterozygosity levels (Peter et al., 2018; Dutta, Dutreux and Schacherer, 2021; Marsit et al., 2021, 2021), and possible differences in the size of adaptive targets, can overwhelm differences in initial fitness between strains, leading to certain genotypes with favorable combinations of “evolvability” traits predictably adapting faster and taking over the population, regardless of their initial fitness.

To empirically test the relative importance of fitness and other traits on long-term success, here we pool together roughly 300 genetically barcoded natural isolates of *S. cerevisiae* and compete them over hundreds of generations and in 20 replicates. The high number of biological replicates allows us to determine how often the fittest strain wins, and how often deviations from this null expectation occur. We perform this long-term competition-evolution experiment in six different environments, so that the fittest strain is different in each case. This allows us to decouple initial fitness from genotype, and ask whether there are environment-specific or strain-specific paths of adaptation, or factors that determine evolutionary success.

We find that initial fitness is, in broad strokes, an excellent predictor of success in this pooled setting. The strains that survive and thrive in our experiment are always drawn from the top 5% of strains, graded by initial fitness in each environment. However, within this high-fitness “nose” of the distribution, we occasionally find that the outcome of the evolutionary competition varies between the replicates of the same environment. It appears that stochastic adaptation and possible frequency-dependent changes in fitness play a role in the heterogeneity of the competition outcomes. We also discover that genomic adaptation often occurs in a strain-specific or environment-specific manner, emphasizing that while evolution may be predictable at certain levels of “coarse-graining,” i.e. fitness classes, there are always idiosyncrasies that are hard to predict deterministically.

## Results

### An experimental system to study the relationship between short-term fitness and long-term evolutionary success in a genetically diverse population

**Fig. 1.**
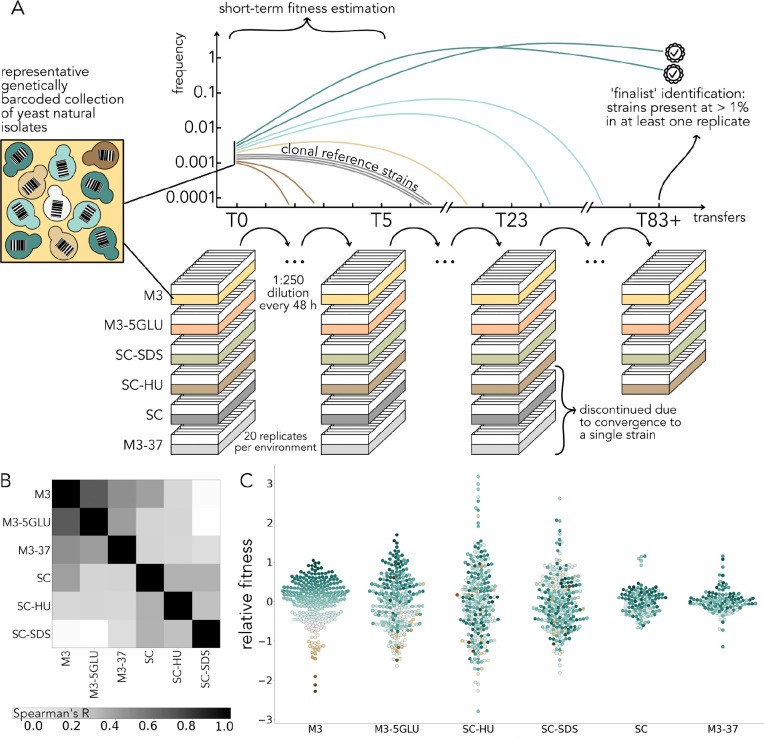
Experimental system to study the relationship between short-term fitness and long-term evolutionary success. **A.** A representative collection of over 300 barcoded wild yeast isolates was propagated in a 10 mL batch culture for over 83 48-hour transfers in 6 different media types and 20 biological replicates per media. Two of the media types were discontinued due to rapid convergence onto a single strain. Inferred strain frequencies from the first five transfers were used to compute short-term fitness and from the last transfer to identify the ‘finalists’ of the evolutionary competition. **B.** Spearman rank order correlation coefficient between fitness values of strains within each environment. **C.** Distributions of relative fitness values in our environments. The colors represent relative fitness in M3.

To empirically measure the extent to which initial fitness predicts long-term success in a natural population we set up a long-term competition experiment using a collection of genetically barcoded wild yeast isolates. This collection consists of over 300 strains and encompasses the phylogenetic, geographic, and ecological diversity of the well-characterized 1011 yeast isolates collection (fig. S1) (Peter et al., 2018). We excluded haploid strains to prevent mating between different isolates, strains with killer virus activity to preclude toxin secretion (Chan, Hays and Sherlock, 2024), and flocculating strains to minimize cell clumping. In addition to the wild isolates, each replicate of our experiment contained 6 barcoded clones of the commonly used haploid S288C lab strain as a reference strain to estimate measurement noise. We propagated barcoded strain pools in batch culture with 1:250 dilution every 2 days in 10 mL of media in 12-well reservoirs without shaking for up to 91 transfers (~720 generations) (fig. 1A). We used six different media types (fig. 1A, table 1), selected to include environment combinations with strong and weak correlations between the initial fitness values (fig. 1B). We had 20 biological replicates per medium to ensure that we sampled the highly probable outcomes of the competition within each environment.

By amplicon sequencing the barcode locus, we constructed barcode frequency trajectories from the first 5 timepoints (fig. 1A, B). We used these trajectories to infer the fitness of each of the natural strains relative to the lab reference strain using a maximum likelihood approach previously developed (Ghosh et al., 2025, Ascensao et al., 2023). Fitness values of the same strain varied dramatically between the environments, with some environments being practically uncorrelated, and some, for example, M3 and M3–5GLU, having similar distributions of initial fitness values (fig. 1B, C). The shapes of the tails of the fitness values distributions also varied across environments, with some environments exhibiting flat tails and others, such as SC-HU, consisting of just three strains that are dramatically fitter than all other strains in the collection.

In two environments, M3–37 and SC, the initially diverse population coalesced to a single strain in all 20 replicates within the first 5 transfers (CGB and CGK, respectively). These strains were initially amongst the fittest in the population in each of these environments and started the competition at the highest frequency. We confirmed that in each of these two environments, the same strain remained dominant up to 23 transfers, or 184 generations, at which point we discontinued these environments (fig. S2, S3).

### Initial fitness is a strong predictor of long-term success

**Fig. 2.**
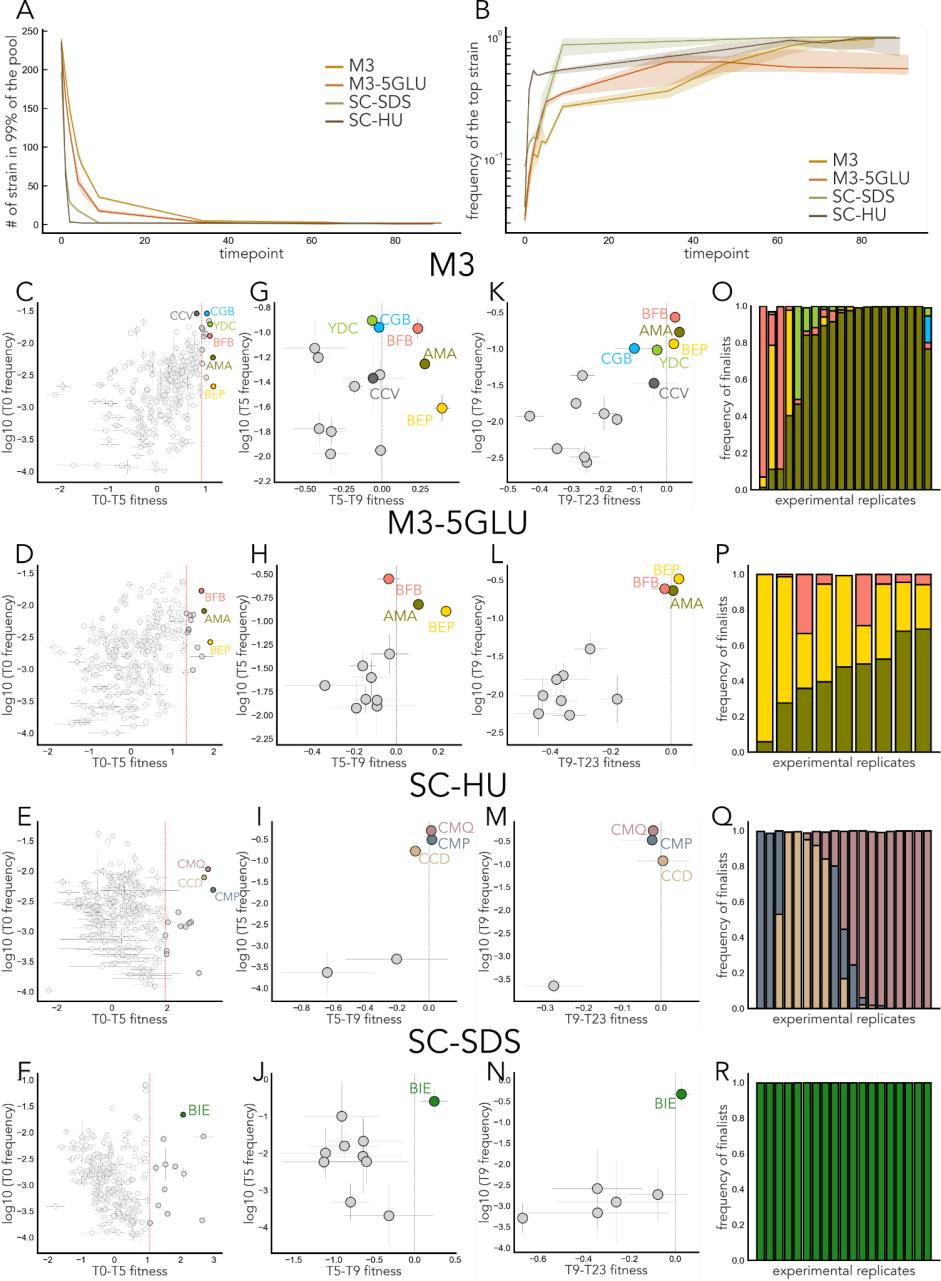
The finalists of the evolutionary competition. **A.** Overall dynamics of the pool composition in the 4 long-lasting environments. The median number of strains composing the majority of the pool (99%) at different timepoints of the long-term evolution experiment and their interquartile ranges. **B**. Frequency of the most abundant strain over time in the 4 long-lasting environments and their standard deviations. **C, D, E, F.** Relative short-term fitness calculated from the first 6 timepoints using the maximum likelihood framework and mean frequency at T0 for all strains in the pool. Colors other than light grey indicate finalist strains. The error bars represent the inferred standard errors. Red dashed lines represent 95th percentile in fitness values. Grey dots represent strains in the top 95th percentile of fitness but not finalists. **G, H, I, J.** Mean change in frequency from timepoint 5 to timepoint 9 for selected strains for the strains in the top 95th percentile. Colors other than light grey indicate finalist strains. The error bars represent standard deviations across biological replicates. Grey dashed lines highlight no change in frequency. **K, L, M, N.** Mean change in frequency from timepoint 9 to timepoint 23 for selected strains. Colors other than light grey indicate finalist strains. The error bars represent standard deviations across biological replicates. Grey dashed lines represent no change in frequency. **O, P, Q, R.** Frequencies of all strains present at ≥ 1% at the last available timepoint for each replicate of the M3, M3–5GLU, and SC-HU environments, respectively. Colors are consistent with the colors from other panels.

At the end of this 6-month experiment, we sequenced the last timepoint for each of the replicates of the remaining four environments, as well as occasional timepoints from the middle of the experiment. Due to unforeseen technical issues, the last timepoint that was available for sequencing varied among replicates within each environment (table 1), and for the M3–5GLU environment, we only had 10 replicates that survived through the end of the experiment. The overall diversity in the pool composition declined at varying speeds in different environments, and the most dramatic changes occurred in the first 10 transfers (fig. 2A, B). Next, we recorded the strains that persisted at high frequency in each environment, and their resulting frequencies within-replicate and within-environment. We define a strain as a “finalist” in a given environment if it was present at ≥1% frequency in at least one replicate at the last available timepoint. No environment had more than five finalists. The SC-SDS environment had the same single finalist in all 20 replicates, and the M3, M3–5GLU, and SC-HU environments had between 3 and 5 finalists, which ended up in different proportions at the final timepoint (fig. 2O–R). Altogether, the long-term dynamics in our experiment revealed a variety of qualitatively different outcomes: clear dominance of a single strain in SC-SDS (fig. 2R), possible long-term coexistence of the same three strains in M3–5GLU, alternative states with one or two dominant strains in SC-HU (fig. 2Q), and a mix of all the above outcomes in M3 (fig. 2O).

**Fig. 3.**
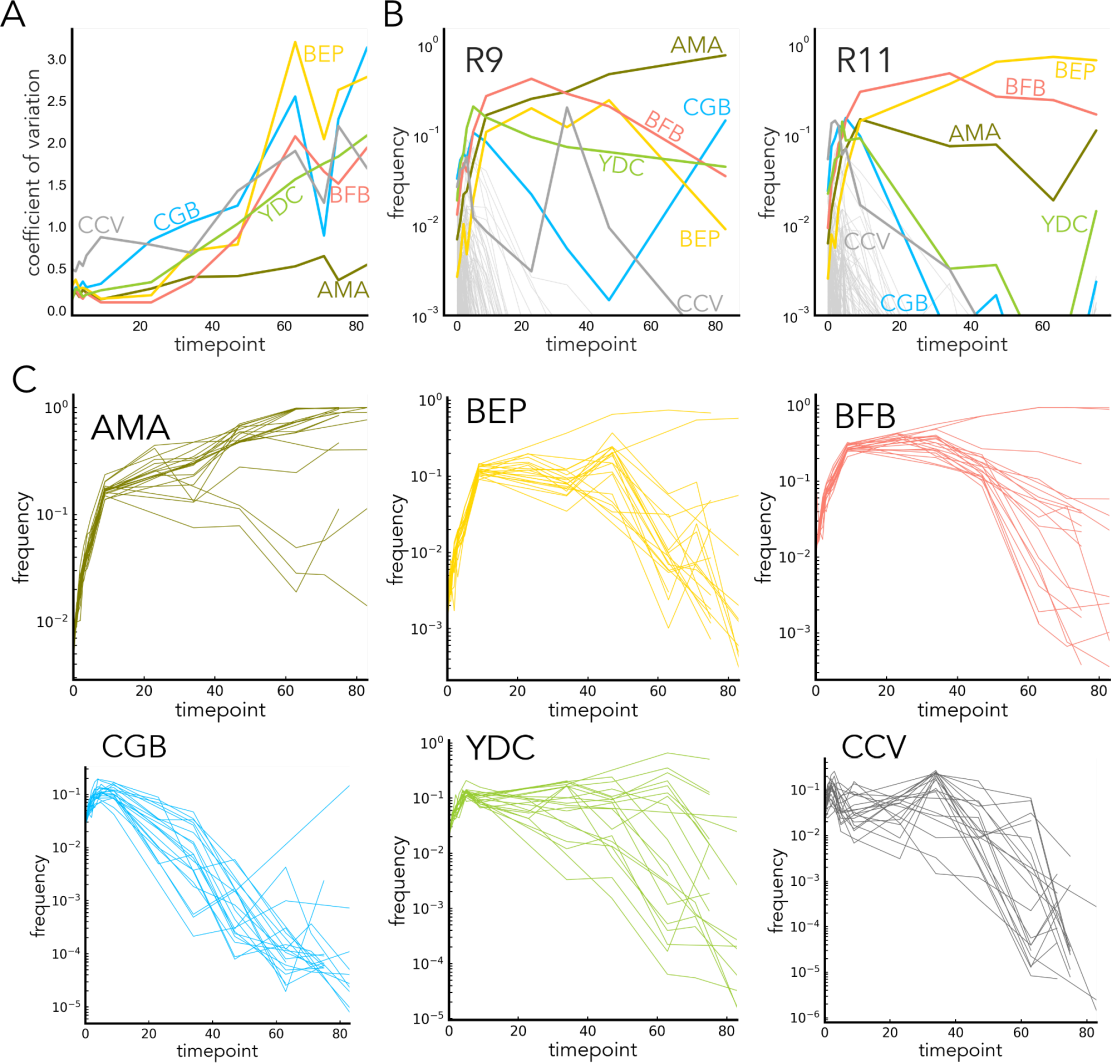
**A.** Coefficient of variance across experimental replicates in the M3 finalist strain frequencies over time. **B.** Two examples of the long-term trajectory in M3. Trajectories for other replicates are in Fig. S4. **C.** Trajectories from the finalist strains in M3 in all replicates.

We reasoned that in the simplest model without evolution of frequency-dependent interactions, the long-term success of a strain is determined purely by its initial frequency and fitness. Therefore, we calculated the mean frequency of all strains at T0 and inferred their fitness from the first six timepoints of the competition using the maximum likelihood method. While the initial frequency of the finalists varied, they were always in the top 95th percentile of initial (T0-T5) fitness in their respective environments (fig. 2C–F). Interestingly, there were occasional strains in the top 95th percentile of the fitness values as shown in fig. 2C–F, but do not persist at high frequency to the end of the experiment in any replicate. We surmised that the reason why these strains never persist at high frequency till the end of the competition might be due to small differences in fitness between them and the finalist strains that cannot be resolved using our fitness measurements from the first 5 timepoints, or in slight changes in the environments due to batch effects or changing strain composition. The trajectories of the finalist strains are concordant during the first ∽30 transfers, which is consistent with competition in the absence of adaptation (fig. 3A, C). Thus, we estimated the frequency of the finalist strains and other strains from the top 95th percentile in the T0-T5 fitness values at T9 and T23 and estimated ‘T5-T9’ and ‘T9–23’ fitness as the frequency change in the strain abundance over these time periods (conditioned that the strains did not go extinct). We observed occasional differences in the rank order between the strains’ fitness values estimated from different timepoints. These differences could be due to the inherent uncertainty of the experimental fitness measurement, to changes in relative fitness as a function of strains’ frequencies, or to small changes in the environment composition. Regardless, the finalist strains tend to cluster in the top-fitness/high-frequency corners of the starting position plots regardless of which time interval we use to estimate the initial fitness. This is not the case for the other strains that cluster with the finalist strains in their starting position (Fig. 2G–N). Therefore, in broad strokes, having a good starting position, especially high initial fitness, is a prerequisite for long-term success.

### Frequency dynamics during long-term competition suggest a role for adaptive evolution

While our initial results suggest that initial fitness is required for a chance of long-term success, the heterogeneity of the outcomes of the long-term competition suggested an influence of stochastic events such as adaptive evolution. Of all our environments, M3 had the most diverse outcomes of the long-term competition. After 10 transfers (approximately 80 generations), only 6 strains are consistently present at frequency above 10^−3^: the five finalists (AMA, BEP, BFB, CGB, and YDC) and another strain, CCV. After about 30 transfers (or ~200–300 generations), the frequency of strains starts diverging (fig. 3A,C, for other environments see S8, S9) and the strains experience occasional flips in rank order, which ultimately results in the heterogeneity of outcomes at the final timepoint. This heterogeneity might arise due to stochastic *de novo* mutations (Kao and Sherlock, 2008), idiosyncratic abundance fluctuations (Ascensao et al., 2025), or, in the simplest null model, due to drift and batch variation.

Remarkably, we occasionally observe what appears to be large-effect adaptive mutations which result in a dramatic increase in frequency. In M3, the most dramatic examples of such sweeps are evident for several replicates of BEP and BFB strains (between transfers 20 and 40) and for one replicate on the CGB strain (between transfers 30 and 50) (fig. 3C). The latter example is particularly remarkable as the CGB strain seemed to acquire the high-effect adaptive mutation while being at below 10^−3^ frequency. Taken together, a closer inspection of the frequency dynamics in our competition revealed hidden complexity behind a simple competition experiment (fig. 3, S4–9). Among the top fitness strains, stochastic adaptive evolution appears to shape the outcomes of a long-term competition. This is consistent with the previous model, suggesting that stochastic large-effect adaptive mutation and clonal interference determine the evolutionary fate of a complex population (Kao and Sherlock, 2008).

### Whole genome sequencing of evolved isolates reveals parallel and divergent paths of adaptation

**Fig. 4.**
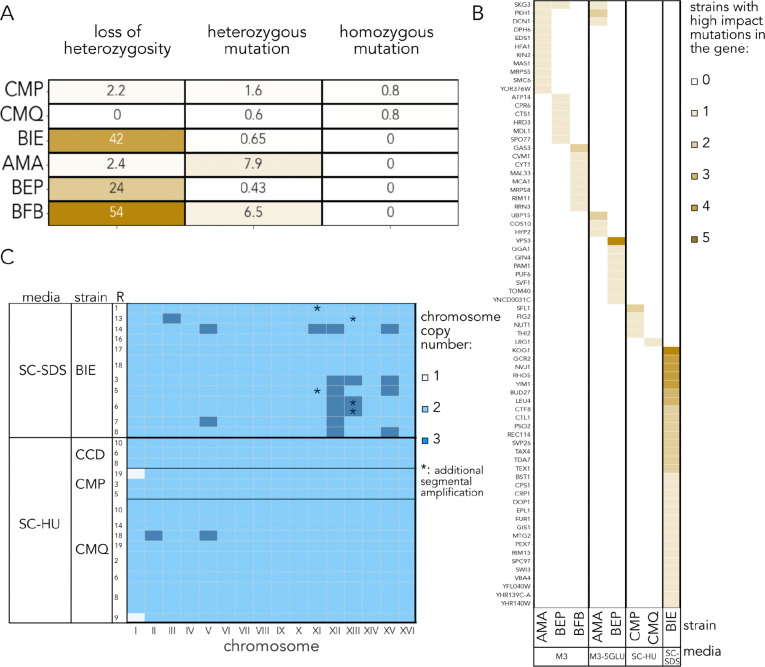
Genetic variation of evolved clones. **A.** Summary of mutation types detected in whole genome sequencing of isolates from evolved populations. Heatmap and annotations represent the average number of variants per number of evolved clones sampled from each strain. **B.** High impact (frameshifts and nonsense) variants in protein coding regions for all environments. Color reflects the number of variants detected in a given gene. **C.** Summary of *de novo* aneuploidies and copy number variation detected in evolved clones. Color represents the number of copies of each chromosome, and asterisk reflects an additional large amplification.

Given that the outcomes of our experiment hinted at the widespread patterns of clonal interference, we wondered what kinds of *de novo* mutations occurred and rose to detectable frequencies during the course of our 6-month competition, and if the identity of the novel mutations could be predicted by the environment, strain background, or both. We isolated potentially evolved clones from the last timepoints in each environment, and whole-genome sequenced them alongside with their ancestral clones. We identified variants that differed between the evolved clones and their respective ancestors, and called *de novo* genetic changes by doing a series of filtering steps described in Methods. Briefly, we filtered out variants that were called with low-quality, were not biallelic, had low sequencing depth, or where the genotype call in the ancestor was unclear. Consistent with adaptive evolution playing a role in our evolutionary competition outcome, we detected substantial genetic variation that was private to the strain isolated from the last timepoint in each evolved clone that we sequenced (fig. 4A). The types of *de novo* mutations varied widely among the sequenced finalist isolates, including aneuploidies and large segmental duplications, loss of heterozygosity (LOH) events, as well as novel heterozygous and homozygous mutations (fig. 4C, S10).

The mutational spectrum depended on both the strain background and environment. For example, aneuploidies and large segmental duplications only occurred in the SC-SDS and SC-HU environments (fig. 4C, S10). The patterns of aneuploidies were unique in each of the evolution replicates (fig 4C). HU causes replication and oxidative stress in yeast (Shaw et al., 2024) and can trigger a variety of mutations, including point mutations, aneuploidies, and structural variants (Li et al., 2023). Aneuploidies occurred in two different strain backgrounds in the SC-HU environment, including an instance of convergent loss of a copy of chromosome I (fig. 4C). However, aneuploidies in the SC-HU environment are likely driven by the mutagenicity of this environment. SDS causes cell wall and oxidative stress (Cao et al., 2020; Zhao et al., 2020), although this was shown only for a dose 4 times higher than the one used in this study. We observed recurrent gains of various chromosomes in the derivatives of the BIE strain, the only finalist in the SC-SDS media. For example, chromosomes 5, 11, 12, 13, and 15 or their parts were amplified in up to 6 independent replicates. Since BIE was the only finalist in SC-SDS it is impossible to know whether the high number of aneuploidies in it is due to the unknown property of SDS to trigger aneuploidy or the impressive robustness of the BIE strain to aneuploidies. However, the latter is unlikely, as the rate of aneuploidies in the unperturbed BIE is low compared to other natural isolates (Dutta, Dutreux and Schacherer, 2022).

Unlike chromosomal abnormalities, the LOH events seem to depend more on the strain background rather than the environment. They occur with high frequency in yeast and play an important role in yeast evolution ((James et al., 2019), reviewed in (Dutta and Schacherer, 2025)). As expected, in our experiment, the LOH events predominantly occurred in the strains with high levels of initial heterozygosity (Table S4) (Peter et al., 2018), and were the primary mode of *de novo* mutation in these strains. Unexpectedly, of the highly heterozygous strains, diploid strains (BEP, BFB, and BIE) appeared to overall have a higher frequency of variants attributed to LOH events than the triploid AMA strain (fig. 4A). This is in contrast to mutation accumulation lines, where ploidy level positively correlates with the LOH rate (Dutta, Dutreux and Schacherer, 2022), although we cannot rule out that this observation is due to decreased statistical power to call heterozygous mutations in the triploid ancestor. Overall, our results suggest that genome architecture shapes the spectrum of *de novo* mutations and thus might influence the adaptive mutation target size.

We then looked for patterns of parallel evolution at the gene level across different strains and environments. Since some of our strains underwent LOH in dozens of kilobases long stretches of DNA, determining which of the individual LOH variants are functionally significant is akin to searching for a needle in a haystack. Therefore, we focused our attention only on protein-coding high-impact mutations such as frameshifts and nonsense mutations. We found several instances of the same loci being targeted among the isolates from different replicates of the same strain within the same environment, meaning that we had enough statistical power to detect such instances of convergence at the gene level. Surprisingly, we saw very few instances of parallel adaptation at the gene level across environments. Fig 4B shows that amongst the genes in which *de novo* variants lie, the only overlap across different ancestral strain backgrounds is in *SKG3*, a protein of unknown function, which has high-impact mutations in both AMA and BEP strains that evolved in M3, as well as AMA from M3–5GLU. There are more instances of apparent parallelism amongst variants which occurred in the AMA ancestor, across different environments. We find that there are high-impact variants in *SKG3*, *PKH*, and *DCN1* in AMA strains that evolved in both M3 and M3–5GLU, suggesting parallelism between these environments both at the level of identity of the finalist strains and at the genomic level.

**Fig. 5.**
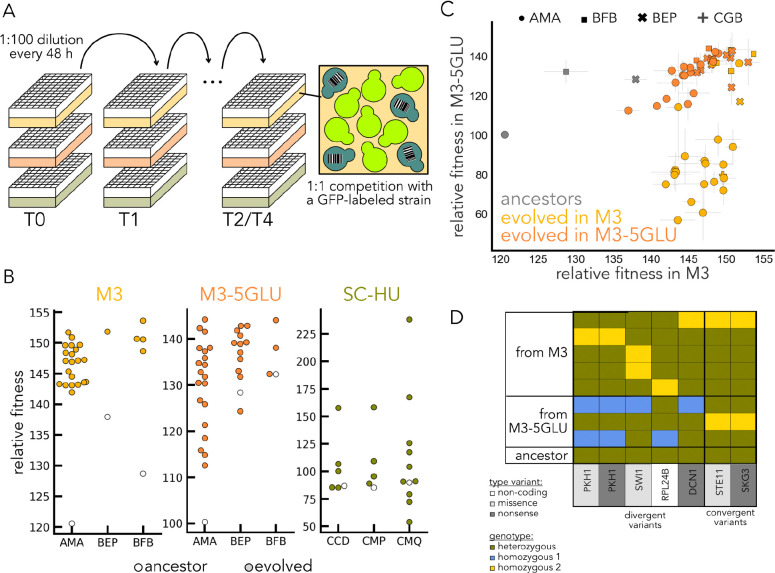
Functional differences in the evolutionary competition finalists. **A.** Individual finalist strains and their corresponding ancestors were cocultured with a GFP-labeled wine strain in 1:1 competitions in 1 mL of media without shaking. We competed the finalists from the M3 and M3–5GLU environments in both of these media, and the finalists from the SC-HU medium in SC-HU. **B.** Relative fitness of each of the strains isolated at the last timepoint relative to its respective ancestor (arbitrary units). **C.** Fitness profiles of the strains isolated from the M3 and M3–5GLU media. Yellow points represent the strains isolated from M3, orange points represent the strains isolated from M3–5GLU, and grey points represent the ancestors. **D.** Variants that co-occur in AMA clones evolved in different environments. Each row is an evolved clone, and colors represent genotypes at different loci. Blue box means genotype is homozygous for allele 1, and yellow box means homozygous for allele 2, and green is homozygous (ancestral).

To examine these putatively parallel events more closely, we analyzed the genotype of each LOH event that occurred at the same locus in evolved AMA strains from M3 and M3–5GLU. Figure 5D shows a summary of the variants that co-occur in both environments on the AMA background. Interestingly, the majority of coincident variants in AMA reverted to different alleles depending on the environment. Unlike most of the other strains in our experiment, AMA is a triploid (Peter et al., 2018), so the ancestor had one copy of one allele and two copies of a second allele. It is not clear if a triploid strain reverts to a rarer allele in one or two steps.

Nonetheless, we find that in five out of seven coincident events, the mutant reverts to a different allele in M3 from M3–5GLU. This suggests that despite the initial similarity of the M3 and M3–5GLU environments (fig. 1B), there are different selection pressures in the two environments. In only two cases do we see heterozygous genotypes converge to the same allele in a LOH event that is coincident across both environments.

Taken together, the timescale of our competition allowed enough time for novel mutations to arise, and the type of these mutations depended both on the environment and strain background. On the genetic level, we detected a few examples of convergence on the level of strain and environment, and a few examples of divergence, including intriguing divergent LOH events in the same site. However, most of the gene targets of adaptation appear to be specific to the strain-environment combination, which suggests that adaptive evolution picks up on small differences between both the genetic background and the environment that are invisible to selection on standing genetic variation.

### Fitness competitions between evolved clones and ancestors reveal functional differences and an asymmetric tradeoff

Whole-genome sequencing revealed substantial *de novo* genomic changes amongst evolved strains from our evolution experiment, with intriguing patterns of parallelism and divergence at the genetic level. We next wondered if these observed *de novo* mutations were adaptive or merely neutral.

By definition, a non-neutral mutation changes some aspect of cellular physiology relevant to fitness in the corresponding evolutionary environment. Thus, if an evolved clone shows a difference in fitness from its ancestor, we can infer that it harbors at least one non-neutral mutation. To test whether this is the case, we performed competition experiments involving the ancestors of the finalist strains and their derivatives isolated at the last timepoint in M3, M3–5GLU, and SC-HU media. Specifically, we competed each strain of interest with a GFP-labeled wine strain UCD595 in a 1:1 competition in 96-well plates (fig. 5A). We found that most of the strains isolated at the last timepoint in our evolutionary competition outperformed their respective ancestor in a competition with a GFP strain (fig. 2C), with only a handful of exceptions. This suggests that the underlying physiology changed for the majority of the finalists of the evolutionary competition, which is consistent with adaptive evolution and clonal interference being the driving forces in determining long-term success in our experiment.

The same three strains – AMA, BEP, and BFB are the finalists in both M3 and M3–5GLU. Since the initial fitness values of the wild strains correlated in these two environments (fig. 1B), it is not surprising that some of the finalist strains are shared. We therefore wondered if the physiological changes induced by the adaptive mutations in these three strains would be convergent on the level of strain, environment, or both. For that, we measured the fitness of each of the derivatives of these strains in both M3 and M3–5GLU environments, regardless of which environment they evolved in(fig. 5C). We found that BEP and BFB strains isolated from either M3–5GLU or M3 improved their fitness in either media. AMA strains isolated from M3–5GLU also improved their fitness in either media. On the contrary, most of the AMA clones evolved in M3 worsened their fitness in M3–5GLU (fig. 5C). This suggests the existence of an asymmetrical trade-off for the evolution in these two environments. Notably, our whole genome sequencing of evolved AMA isolates revealed a divergence at the genetic level between strains that evolved in M3 and M3–5GLU. We found that even when the same genetic locus was mutated, the variant often reverted to a different allele depending on the evolution condition (fig. 5D). This suggests that at least for the AMA strain, evolution in the M3 and M3–3GLU proceeds qualitatively differently and leads to divergent physiological changes.

Taken together, the fitness re-measurement against a GFP-labeled strain confirms that the natural isolates evolved non-neutrally during our competition. These results also reveal an asymmetrical trade-off among the derivatives of the AMA strain isolated from the M3 media.

## Discussion

In this study, we experimentally tested to what extent initial fitness determines long-term evolutionary success of a genotype. We used a genetically barcoded pooled collection of wild *Saccharomyces cerevisiae* isolates to track strains as they compete and evolve in real time. This naturally diverse collection likely encompasses the differences in most variable traits, including any possible evolvability traits, i.e., traits that determine an organism’s ability to adapt in the future. Thus, we reasoned that if standing genetic variation in such evolvability traits is more substantial than the standing genetic variation in fitness, we would see that the strains’ performance in our evolutionary competition has little correlation with their starting fitness.

We found that in all six of the evolution environments in our experiment, the initial fitness was the most significant predictor of long-term success (fig. 2). However, among the top fitness strains, there was a heterogeneity in the long-term competition outcomes (fig. 2O–R), and the dynamics of the strains’ frequency changes are consistent with occasional high-effect adaptive mutations and clonal interference. In addition, we cannot rule out possible frequency-dependent changes in fitness of the finalist strains, as for some of the strains there are detectable differences in their T0-T5, T5-T9, and T9-T23 fitness values.

Consistent with adaptive evolution playing a role in our experiments, the whole genome sequencing of the evolved clones revealed *de novo* mutations that arose in the course of our competition. Interestingly, the predominant type of novel mutations depended both on the environment and the generic features of genome architecture such as ploidy and heterozygosity. On the contrary, specific gene targets of adaptation were unique to the combination of strain and environment. Especially surprising to us was the virtual lack of convergence between the shared finalist strains in the M3 and M3–5GLU media. These two environments are generally similar (fig. 1B) and the shared three strains mean that broadly the same traits are selected for during the initial steps of evolution. However, the gene targets of *de novo* mutations (fig. 4A) and the physiological differences between the evolved clones and their ancestors (fig. 5C) are determined by both strain and environment. Occasionally, these environment-strain-specific mutations result in a trade-off. For example, some of the derivatives of the AMA strain, originally very fit in both M3 and M3–5GLU environments, lost fitness in M3–5GLU after evolution in M3. It is not clear what the genetic determinants of this trade-off are. One exciting possibility is that it might be due to divergent LOH events in this strain, where an initially heterozygous allele reverted to different homozygous alleles in the two different environments. Given that the AMA strain is initially a triploid and reversion to a rarer allele in a triploid is likely a rare event, such divergent LOH is likely adaptive. If proven to be so, divergent LOH constitutes a mechanism for the generation of mutations that carry trade-offs.

While this study makes significant progress in investigating the role of short-term fitness in long-term evolutionary success, it does not come without limitations. First, even though we picked our environments to maximize the diversity of the initial fitness values between the strains, having only six environments means that strains that are not among the top fit strains in any environment are driven to extinction within the first few transfers, having almost no chance to exhibit evolvability traits. Second, while having 20 replicates was enough to draw the initial conclusion about the role of short-term fitness in long-term success, this sample size is not enough to detect small differences in evolvability. Further evolution experiments specifically competing a small number of strains in a large number of replicates are necessary to overcome this limitation. Lastly, the concrete measurements of the adaptive mutation target size and rate is currently missing for most strains in our collection. However, such measurements are needed for precise measurements of likely small, naturally occurring evolvability differences.

To conclude, we demonstrated that initial fitness is a strong but not perfect predictor of evolutionary fate. We showed that both environment and generic genome organization features, such as ploidy and heterozygosity level, shape the spectrum of *de novo* mutations in an evolving organism. Finally, we show that the evolutionary paths of physiologically similar and genetically related organisms could be highly specialized, with little overlap on the genotype level.

## Materials and Methods

### Barcoding wild yeast collection

The wild yeast isolates used in this experiment (table S3) are a subset of the 1011 wild yeast isolates collection (Peter et al., 2018). This subset is composed of strains with unique DNA barcodes integrated into their genomes, to enable pooled assays (manuscript in preparation from Schacherer lab). For our pooled evolution competition, we first excluded from the barcoded collection strains with duplicate genetic barcodes and strains that did not grow after thawing a glycerol stock. Additionally, we excluded haploid, flocculating, and killer strains from the barcoded wild isolates collection.

Haploid strains are a minority in the wild isolates collection, and to prevent mating between different isolates we decided not to include any haploid strains in our experiment. To test for haploids, we used a modified flow cytometry and SYBR green protocol to measure DNA content (Dunham, Gartenberg and Brown, 2015). Individual strains in the collection, as well as known haploid and diploid control strains were grown in YPD to saturation overnight and then diluted and grown for ~5 hours to exponential phase in deep-well 2mL assay blocks. At this point, cultures were pelleted and resuspended and incubated at room temperature for 1 hour in 1mL of 70% ethanol. Cells were then washed and incubated in 0.2 mg/mL RNAse A solution and at 37°C for 2–4 hours to digest RNA and then washed and incubated at 50°C in 2mg/mL proteinase K for 1 hour. After digestion steps, cells were resuspended into FACS buffer (200mM Tris-Cl pH 7.5, 200mM NaCl, 78mM MgCl_2_). 200 *μ*l of SYBR green diluted 5000X was added to each tube and mixed vigorously to reduce clumping and stored in the dark where possible. Samples were then run on the flow cytometer. We recorded at least 10000 events per strain and used the ‘BL1-H’ channel to estimate DNA content in each of the cells. We normalized the values to known haploid and diploid strains and assigned each of the strain ploidy of 1, 2, or higher ploidy value. We excluded all the strains that scored as haploids from our pooled collection.

To measure flocculation in the wild isolates, we used a modified protocol from (Yone et al., 2022). We first inoculated glycerol stock of each strain into 1mL of YPD media and grew them overnight in 96-well deep well plates at 30°C with no shaking. Next day, we used the Integra Viaflo 96 to first mix the overnight cultures and transfer 500 *μ*l of the cells into a new 96-well deep well plate. Then, we mixed these cultures by pipetting up and down 30 times at the highest speed, removed the pipette tips and waited for exactly 1 min. Next, we transferred the top 100 500 *μ*l of the cells into a new 96-well plate. In 10 sec, we added 100 *μ*l of 10 mM EDTA to the bottom fraction, mixed 30 times, and transferred 100 *μ*l of the bottom-cell-fraction-EDTA mix into a new 96-well plate. Immediately after the transferring of the top and bottom fraction cells into their respective 96-well plate, we measured the optical density of these samples at 600 nm using a BioTek Epoch2 platereader. We then subtracted the blank media OD measurement from every sample and calculated flocculation score for each strain using the formula: flocculation score =1 - OD_top cell fraction_ / OD_bottom cell fraction_ · 0.8. We confirmed high flocculation status for some of the known flocculators in the collection and additionally discovered 4 more flocculating strains. We did not include any strain that was either labeled as a flocculant in the collection metadata or scored as a flocculant in our essay into our experiment.

To identify wild strains producing killer toxin, we screened all barcoded natural isolates for K1 killer toxin production using a lawn-based assay adapted from (Crabtree et al., 2019). Assays were performed on YPD agar buffered to pH 4.6 with sodium citrate (final concentration 0.1M ). After autoclaving, 10 mL of methylene blue solution (final concentration 0.003% w/v) was added per liter as a marker of oxidative stress to enhance halo detection. Agar (50 mL) was poured into single-well rectangular dishes to ensure compatibility with a 96-well pipetting robot. Barcode and Lawn strains were revived from −80 °C glycerol stocks by streaking onto Sabouraud agar plates. A single colony of each barcoded strain was inoculated into 1 mL of Sabouraud broth in 96-well plates and incubated for 48 h at 25 °C with shaking (200 rpm) to saturation. A known “super-killer” strain (GSY1057) was included in one plate as a positive control. Cultures were maintained at 25 °C because killer toxins are heat-labile. Lawn cultures were prepared by inoculating 5 mL of Sabouraud broth in 25 mL flasks, incubating for 48 h at 25°C, and diluting 1:50 before plating. For each assay plate, 500 μL of diluted lawn culture was spread evenly across the agar surface. The primary lawn strain was a killer-sensitive BY-background strain (S) from (Pieczynska et al., 2016). As controls, we included the K1 killer strain (K) as a resistant lawn and an uninoculated mock lawn to monitor contamination and assess isolate growth in the absence of competition. For the assay, all test cultures were diluted 1:100, and 5 μL of each strain was spotted onto plates containing one of the three lawn types. Spots were applied in alternating positions, leaving empty wells between samples to allow sufficient space for halo detection. Plates were incubated at 25 °C for 5 days, after which halo formation was scored by visual inspection. All strains grew on the mock lawn. Strains that produced visible halos on sensitive lawns were recorded as killer-positive.

To pool the selected strains, we first streaked each of the barcoded wild strains to single colonies on a YPD plate, inoculated one colony into liquid 1 mL YPD media in a 96-deep-well plate, sealed with a ‘Breath-EASIER’ seal (Cat#: BERM-2000), and grown till saturation for 2 days at 200 rpm shaking at 30°C. After that, we pooled equal volumes of each strain together, mixed, and saved at −80°C as 500 ul glycerol stocks.

### Barcoding reference strain

To barcode lab strain and make it compatible with the barcoded natural isolates collection, we first amplified a fragment from the plasmid carrying a HygR gene, pSK26 (plasmid map included in the supplement), using primers SK168 and SK169 (table S1). The resulting fragment contained the *HYG* resistance marker flanked by 20 bp random barcodes and regions of homology to the *HO* gene. We then transformed haploid strain GSY145 (S288C mat alpha, *ho*) (Kao, Schwartz and Sherlock, 2010) with this fragment. To validate the transformation, we amplified the DNA from the hygR clones with primers SK144/145 (table S1) and Sanger sequenced the resulting 291 bp fragment to identify the barcodes of the newly generated barcoded strains (table S2).

### Evolution media

We prepared all evolution media based on M3 or SC pre-defined media in batches of 4L at once (∽20 transfers per batch) and distributed them in the wells of 12-deep-well plates in a volume of 10 mL at least 2 days before use to detect possible contaminations before the transfers. We stored media at room temperature except for the SC-HU media, which we stored at 4°C. During the growth, we sealed the plates with a ‘Breath-EASIER’ seal (Cat#: BERM-2000) and incubated them at 30°C without shaking.

### Media

To make media for the long-term competition, we first prepared the base media, M3 or SC in batches of 12 L following the recipes below. Then, we added the required amounts of the add-on drugs as needed for the specific media type (Table 2). We filter-sterilized the solutions and pre-poured them into the 12-well plates for storage and contamination control. We stored all media at room temperature except for the SC-HU media, which we stored at 4°C.

M3 (1L) adopted from (Verduyn et al., 1992):

- glucose: 15 g
- potassium phosphate monobasic: 15 g
- MgSO4*(H2O)5: 2.5 g
- ammonium sulphate: 40 g
- vitamin mix (biotin 0.05 g, calcium pantothenate 1 g, nicotinic acid 1 g, inositol 25 g, thiamin HCl 1 g, pyridoxine HCl 1 g, para-aminobenzoic acid 0.2 g, 1000 mL H2O pH 6.5): 5 mL
- minerals mix (EDTA (15 g EDTA, 4.5 g ZnSO4.7H2O, 1 g MnCl2.4H20, 0.3 g CoCl2.6H2O, 0.3 g CuSO4.5H20, 0.4 g Na2MoO4.2H2O, 3.4 g CaCl2, 3 g FeSO4.7H2O, 1 g H3BO3, 0.1 g KI, 1000 mL H2O, pH 4.0): 5 mL

SC (1L):

- YNB 6.7 g
- complete dropout 2 g
- glucose 120 g
- H2O 100 mL

### Evolutionary competitions

We first inoculated 500 *μ*l of glycerol stock containing the barcoded wild yeast pool into 80 mL of YPD media supplemented with 100 μg/mL carbenicillin to prevent bacterial contamination, and the barcoded reference strains into 5mL each and incubated for 1 day at 200 rpm and 30°C. Next day, we created subpools of reference strains (combinatorial combinations of strains which were unique for each replicate and media in our experiment) by mixing together equal amounts of each strain. Then, we added the wild yeast pool to each of the reference subpools in proportion 9:1 (wild yeast):(reference yeast). Finally, we transferred 40 *μ*l of the resulting pools with both wild and reference strains into 10 mL of the corresponding evolution condition media to initiate the timepoint 0 of our evolution experiment. We also saved aliquots of each of the pools in glycerol and sorbitol solutions for further DNA extractions.

From then on, we performed a transfer once every 48 hours by first carefully mixing the evolution media until the settled yeast became distributed evenly across the volume of the media. We then transferred 40 *μ*l of the cell suspension into fresh media. We saved a 100 *μ*l aliquot of the suspension in 16% glycerol solution at −80°C and and a 1mL aliquot in sorbitol solution (0.9 M sorbitol, 0.1 M Tris-HCL pH 7.5, 0.1 M EDTA pH 8.0) at −20°C every 5 transfers, except for the first 38 transfers when we saved an aliquot in sorbitol every transfer. We continued the experiment for between 23–91 transfers, depending on the evolution media.

### DNA extractions

We extracted DNA from 96-well plates using a custom column purification method. Briefly, we first thawed sorbitol-saved cells, spun them down, and discarded the supernatant. Next, we resuspended the cell pellets in 400 *μ*l tissue lysis buffer (NEB, cat# T3011L) supplemented with 7*μ*l of 10 mg/mL RNase A (Sigma, cat# R5000–1G/50–177-9327) and 1.2 *μ*l of 20 U/*μ*l lyticase (Sigma, cat# L4025–1MU), transferred the lysis buffer-cell mix to a deep well plate prefilled with 100 *μ*l of 500 *μ*m acid-washed glass beads (cat# G8772–1KG), and incubated at 37°C overnight. The next day, we vortexed the lysis buffer-cell-bead mix for 5 min at the highest speed in a bead-beater, incubated at 65°C for 15 minutes, spun down the plates for 10 min at 3700 rpm, and transferred the supernatant to a DNA purification column (cat# BD96–01). We washed the column twice with a wash buffer (NEB, cat# T3015–2) and eluted the extracted DNA into 40 *μ*l of elution buffer (NEB, cat# T3016–2).

### Amplicon library preparations

To amplify the barcode for next-generation sequencing, we performed a two-step PCR protocol. For the first PCR step, we mixed 1 *μ*l of extracted DNA, 0.75 *μ*l of the forward and reverse 1st step primers at 10 *μ*M concentration (table S1), 7.5 *μ*l of the NEBNext^®^ Ultra^™^ II Q5^®^ Master Mix (cat #M0544X), and 5 *μ*l of ultrapure water (Invitrogen, cat # 10977–015). The first step primers include a variable index region which allows for demultiplexing of the samples. We run the following PCR program:

Lid: 105°C, volume: 15 *μ*l

1. 98°C, 30 sec
2. 98°C, 30 sec
3. 66°C, 30 sec
4. 72°C, 30 sec
5. go to step 2 and repeat 35x times
6. 72°C, 5 min
7. 4°C, hold

After validating the correct size of the samples and the absence of amplification in the no-template control, we performed the second PCR step. First, we diluted the 1st step PCR product by 1000 fold using the ultrapure water. We then mixed 6 *μ*l of this diluted PCR product, 0.75 *μ*l of the forward and reverse 2nd step Illumina primers (Dual Indexed i5 and i7 primers), and 7.5 *μ*l of the master mix (NEB, cat #M0544X). We run the following PCR program:

Lid: 105°C, volume: 15 *μ*l

1. 98°C, 30 sec
2. 98°C, 30 sec
3. 62°C, 30 sec
4. 72°C, 30 sec
5. go to step 2 and repeat 11x times
6. 72°C, 5 min
7. 4°C, hold

After validating the correct size of the samples and the absence of amplification in the no-template control, we pooled the libraries together and purified using the Monarch PCR & DNA Cleanup kit (#T1030L), and sequenced on a NovaSeq lane.

### Barcode extraction pipeline

To extract and count DNA barcodes from Illumina sequencing reads, we first merge the forward and reverse reads using PEAR. We then demultiplex the reads further by the indices contained in primers from the first step of PCR. To do this, we use regular expressions based on best practices recommended by current literature (Johnson, Venkataram and Kryazhimskiy, 2023). We identify the constant regions using a template of the read, and we extract the forward and reverse indices, and the barcode, to classify the read by sample and barcode. Last, we align the barcode region to a reference template and determine the strain identity. We count reads for each barcode in each sample to quantify barcode frequencies across time. Source code is available at https://github.com/omghosh/longterm-natties.

### Fitness estimates

We estimate initial fitness according to the maximum-likelihood method described in Ghosh et al., 2025, adapted from (Ascensao et al., 2023). This method is described in detail in (Ghosh et al., 2025), and source code is available at https://github.com/omghosh/limiting-functions.

Briefly, we treat the read count of a particular barcode as a negative binomial random variable, and use maximum likelihood inference to estimate the initial frequency of the barcode, and the selection coefficient, or fitness value, from the entire frequency trajectory, under a standard model of frequency change and exponential growth. We infer the noise and standard error on our estimation, as well as the mean fitness of the population, from the reference strain with multiple barcodes. We assume that the log fold change in frequency of a barcode compared to the reference strain, corrected for the changing mean fitness of the population, is equivalent to its relative fitness.

For later time interval fitness estimates, we simply use the log fold change in frequency of individual barcodes between the two time points, which reflects the fitness of these strains relative to the mean of the population.

### ‘Finalist’ clone isolation

To isolate the ‘finalists’ of the long-term competition, we first revived the glycerol stocks from the last available timepoint for each media type in 1 mL of YPD media and grew them overnight. Next, we plated the overnight cultures on solid YPD media. When the colonies appeared, we isolated 2 individual colonies from each timepoint of each media type, and used them to grow new overnight cultures. We saved these cultures as glycerol stocks and used them for further DNA extractions and follow-up experiments.

### ‘Finalist’ clone whole genome sequencing

To sequence the ‘finalists’ of the long-term competition, we first inoculated their glycerol stocks into 1 mL of YPD media and grew overnight. Next day, we spun down the cultures and proceeded with DNA extraction as described above. We then used the transposome tagmentation method (Illumina DNA Prep kit, cat #20060060) to fragment the DNA and amplify libraries for sequencing.

### Whole genome sequencing analysis

First, because our evolved clones came from multiple different strain backgrounds, we obtained reference genomes from http://1002genomes.u-strasbg.fr/files/1011Assemblies.tar.gz and downloaded all assemblies (Peter et al., 2018). We merged all fastq files from the same sample (because we sequenced several samples multiple times) into a single fastq files. We used bwa to align the reads to each reference genome and generated gvcf files. We merged all gvcf files into vcf files for all samples with the same reference genome (i.e. same ancestral strain). Lastly, we used bcftools to generate a csv file from each vcf file, which allowed us to do downstream filtering and analysis on the called variants. Source code is available at https://github.com/omghosh/longterm-natties.

To filter the variants, we first removed all multiallelic variants, the variants that cannot be confidently called in the ancestral strains, the variants with coverage less than 5 or more than double of the median coverage of the sample, and genotype quality of less than 99. We additionally removed all variants that mapped to mitochondria, yeast plasmids, telomeres, ribosomal genes, mobile genetic elements, centromeres, and pseudogenes. For each strain, we called a variant a new heterozygous mutation if its allelic frequency in the ancestor and other strains was < 0.2 or > 0.8. Likewise, we called a variant an LOH variant if its allelic frequency is > 0.2 and < 0.8 in all strains but the strain of interest. To annotate each of the identified variants, we ran SnpEff (Cingolani et al., 2012) command using the R64 version of the *Saccharomyces cerevisiae* S288C genome: *snpEff -dataDir /snpEff_data -v -canon R64-5-1 variants_filtered.vcf > variants_filtered.ann.vcf*.

To detect aneuploidies and other large chromosomal abnormalities, we first aligned all of the sequenced reads to the reference genome. Next, we ran the Samtools (Danecek et al., 2021) command ‘*samtools depth -a input_merged.sorted.rg.coord.bam > output_.txt*’ to obtain coverage for each of the bases in the reference genome. For samples with the median coverage of more than six, we calculated median coverage per chromosome, normalized it to the median coverage of the sample, and rounded it to the nearest 0.5. To visualize the genome-wide coverage, we calculated the mean coverage depth for each kilobase of the genome sequence and plotted it against the genome coordinates.

### Cloning of the pRCC_N9 plasmid with the GFP and NatR gene

To create a pRCC-N9 plasmid, we amplified a 4704 bp fragment from the plasmid pRCC-N (Generoso et al., 2016) using primers SK18 and SK67 and a 1869 bp fragment from the plasmid pGS62 (Kao and Sherlock, 2008) using primers SK72 and SK73. We purified both fragments using a column purification kit (NEB, cat #T1130L) and digested with DpnI (NEB, cat #R0176S) to eliminate the unamplified plasmid. We then performed a Gibson assembly reaction with both PCR fragments (NEB Cat #E2621S), transformed into *E. coli* (NEB Cat #C3019H) and validated successful clones by sequencing. The plasmid map is available in the supplement.

### Cloning of a GFP-labeled yeast strain for FACS-based fitness measurement

We GFP-tagged an all-purpose commercial diploid wine strain UCD595. We first amplified a fragment from the plasmid pRCC-N9 using primers JV_P2_For and JV_P2_Rev. The resulting fragment contained the NAT resistance marker, GFP under the *ACT1* promoter, alongside regions of homology to the *HO* gene. We then transformed the UCD595 strain with this fragment. To validate the transformation, we amplified DNA from natR clones with the primers JV_P3_For and JV_P3_Rev (table S1). We screened three separate transformants for GFP expression in a flow-cytometer (Attune NxT cytometer) and proceeded with one transformant in which >99% detected cells displayed detectable levels of GFP. This transformant is labelled as JV104.

### Cytometry-based fitness measurement of the evolved clones

We inoculated the selected wild isolates, their evolved derivatives, and a GFP-labeled strain JV104 from glycerol stocks and grown without shaking in 1 mL of a 96-well plate filled the measurement media for two 48-h transfers of 10 *μ*l. One day later, we mixed the cells in 1:9 ratio (strain of interest:JV104) by volume and took a 20 *μ*l aliquot for cytometry analysis. A pure JV104 strain and pure version of each of the selected wild isolated was used a control, and each measurement was performed in two biological replicates. On the second day, we performed a 10 *μ*l transfer into a fresh media. After that we continued sampling 20 *μ*l aliquots for FACS measurement on every odd day and transferring the cells in a fresh media on every even day for 1 to 3 transfers. The collected cells for cytometry analysis were stored in 200 *μ*l of PBS solution (Invitrogen, #AM9624) at 4°C for up to 5 days. Each sample was analysed for at least 10000 events on a Attune NxT cytometer using the ‘BL1-H’ filter. Singlets were selected by sorting out any particles with a ratio of forward scatter area and height less than 0.9 or more than 1.03. We then computed the percentage of fluorescent cells for our control samples with 100% GFP and 100% dark cells of each or our wild isolated. Since not all of GFP cells fluoresce and some of non-GFP cells exhibit weak fluorescence, we used these control cultures to calibrate the thresholds of the ‘SSC-A’ and ‘BL1-H’ parameters. We then applied these thresholds to each of our samples and calculated the percentage of GFP cells in each of them. By subtracting the percentage of GFP cells from 100 we obtained the fraction of each of out strains at each timepoint in the competition with the JV104 strain. We visualized the results by plotting the trajectories of each stain over time and calculated their relative fitness values by computing the areas under the curves for each strain.

## Supplementary Material

Supplement 1

Supplement 2

## Figures and Tables

**Table 1.** Environments used in this project

environment nickname	base media	glucose %	incubation temperature (°C)	added drugs	replicates	last sequenced transfer
M3	M3	1.5	30	none	1–10	83
					11–20	75
M3-37	M3	1.5	37	none	1–20	23
M3-5GLU	M3	5	30	none	1–10	91
SC	SC	2	30	none	1–20	23
SC-SDS	SC	2	30	sodium dodecyl sulfate (SDS, 0.0075% final concentration)	1–10	88
					11–20	79
SC-HU	SC	2	30	hydroxyurea (HU, 3 mg/mL final concentration)	1–10	89
					11–20	79

**Table 2.** Media for the long-term competition

evolution media	base media	add-ons	incubation temperature (°C)
M3	M3	-	30
M3-37	M3	-	37
M3-5GLU	M3	extra glucose (5% final concentration)	30
SC	SC	-	30
SC-SDS	SC	SDS (0.0075% final concentration)	30
SC-HU	SC	hydroxyurea (3 mg/mL final concentration)	30
