# Supplementary material for: An empirical long-term competition among natural yeast isolates reveals that short-term fitness largely but not entirely predicts long-term outcomes
Source: bioRxiv. 2025 Oct 10:2025.10.09.681448. Preprint. [Version 1] doi: 10.1101/2025.10.09.681448 (PMC12632622; doi:10.1101/2025.10.09.681448)
Supplement: Supplement 1 [file media-1.pdf]

LT-EVO PAPER SUPPLEMENTAL FIGURES AND TABLES

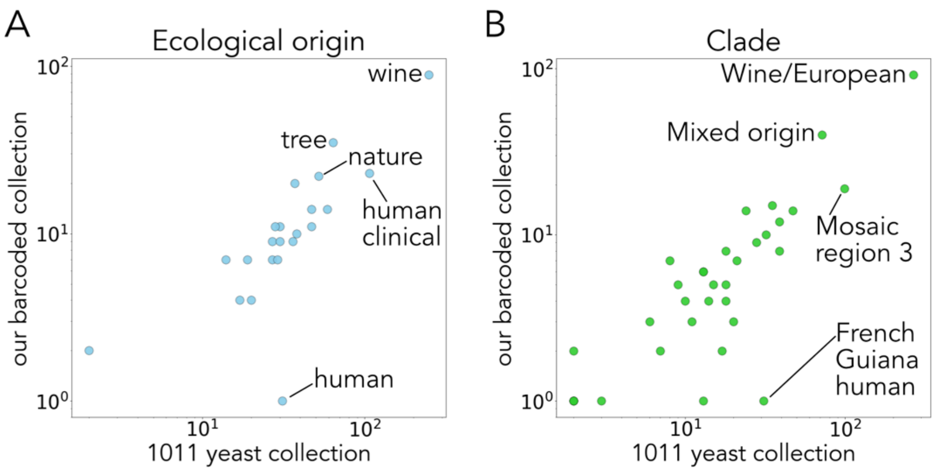

Fig. S1. Comparison of our barcoded wild yeast collection and the benchmark 1011 wild yeast collection. **A.** Representation of ecological origin in both collections. **B.** Representation of clades in both collections.

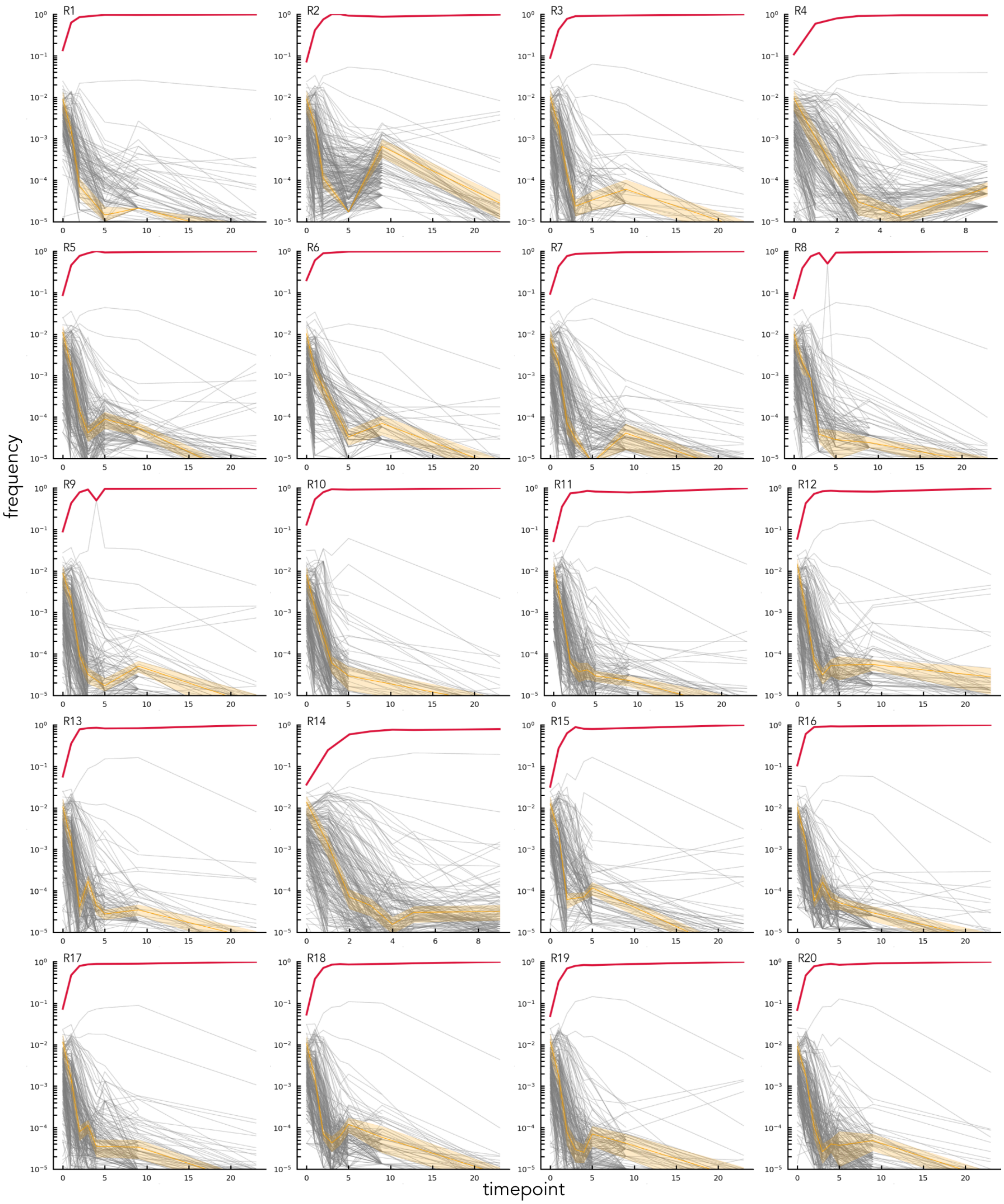

Fig. S2. Convergence to 1 strain in the M3-37 environment. Red lines are the CGB strain, yellow lines are the reference lab strains, and all other strains are in grey.

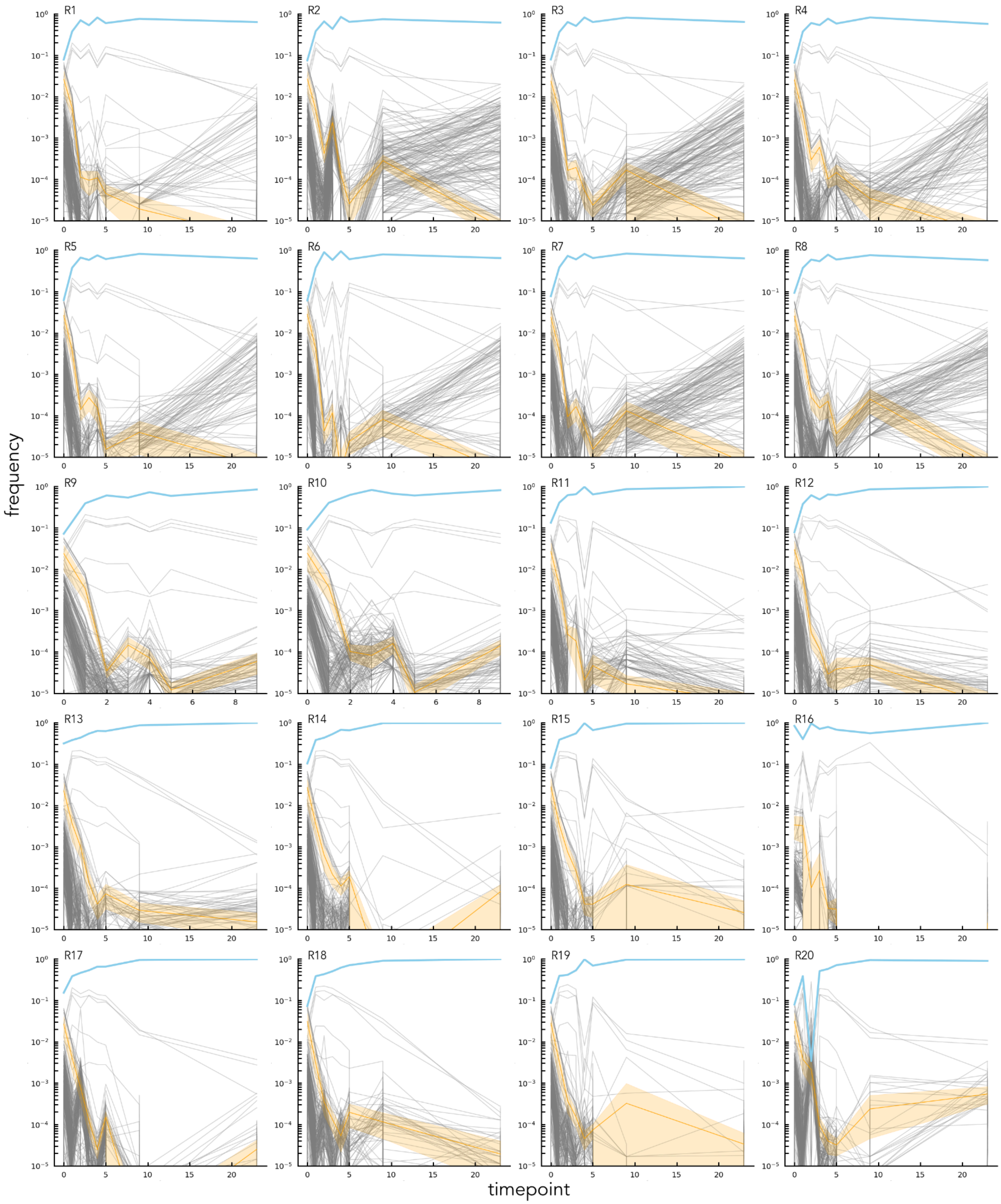

Fig. S3. Convergence to 1 strain in the SC environment. Blue lines are the CGK strain, yellow lines are the reference lab strains, and all other strains are in grey.

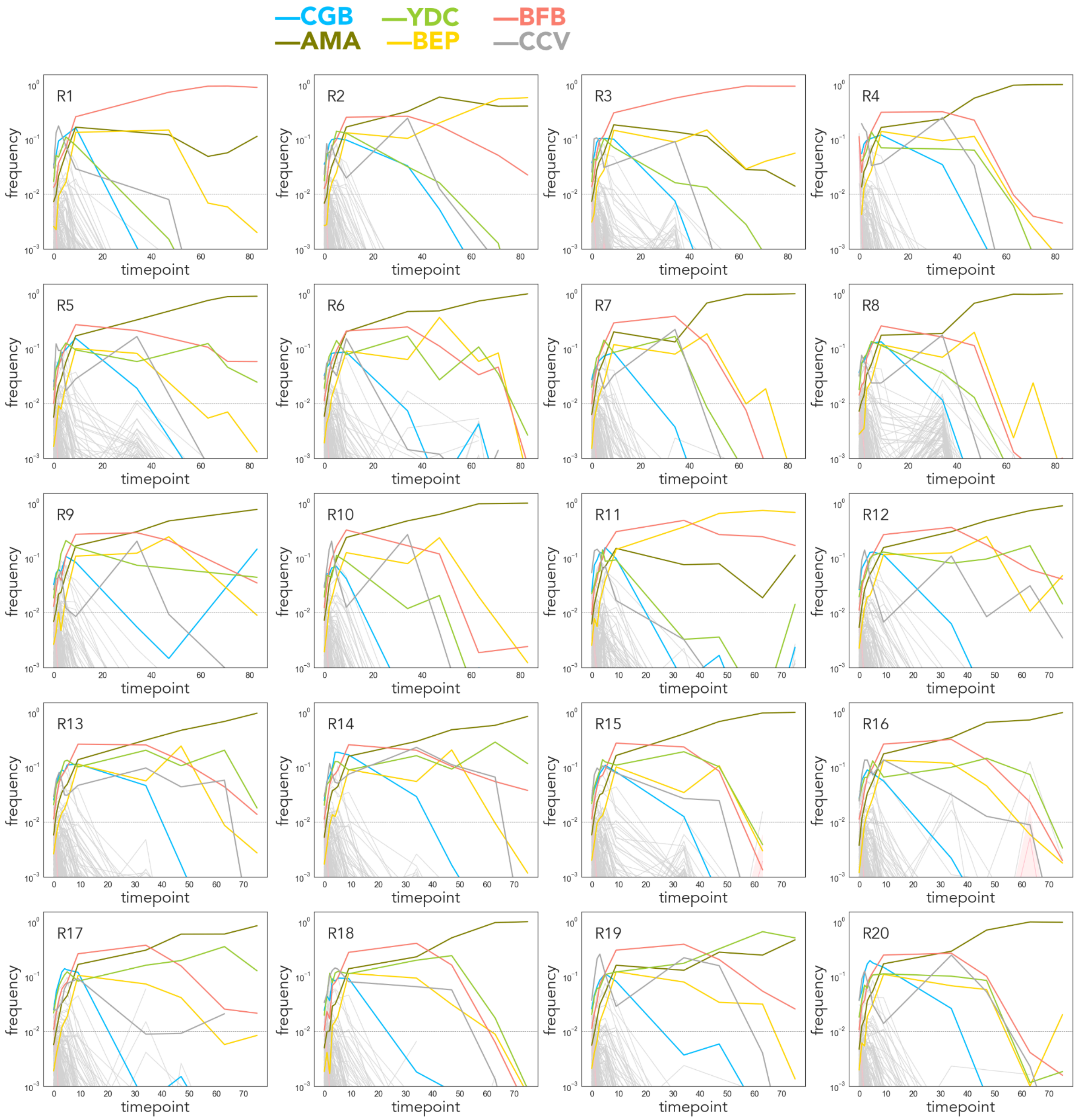

Fig. S4. Trajectories of the strains in the M3 environment with the finalist strains and the CCV strain highlighted.

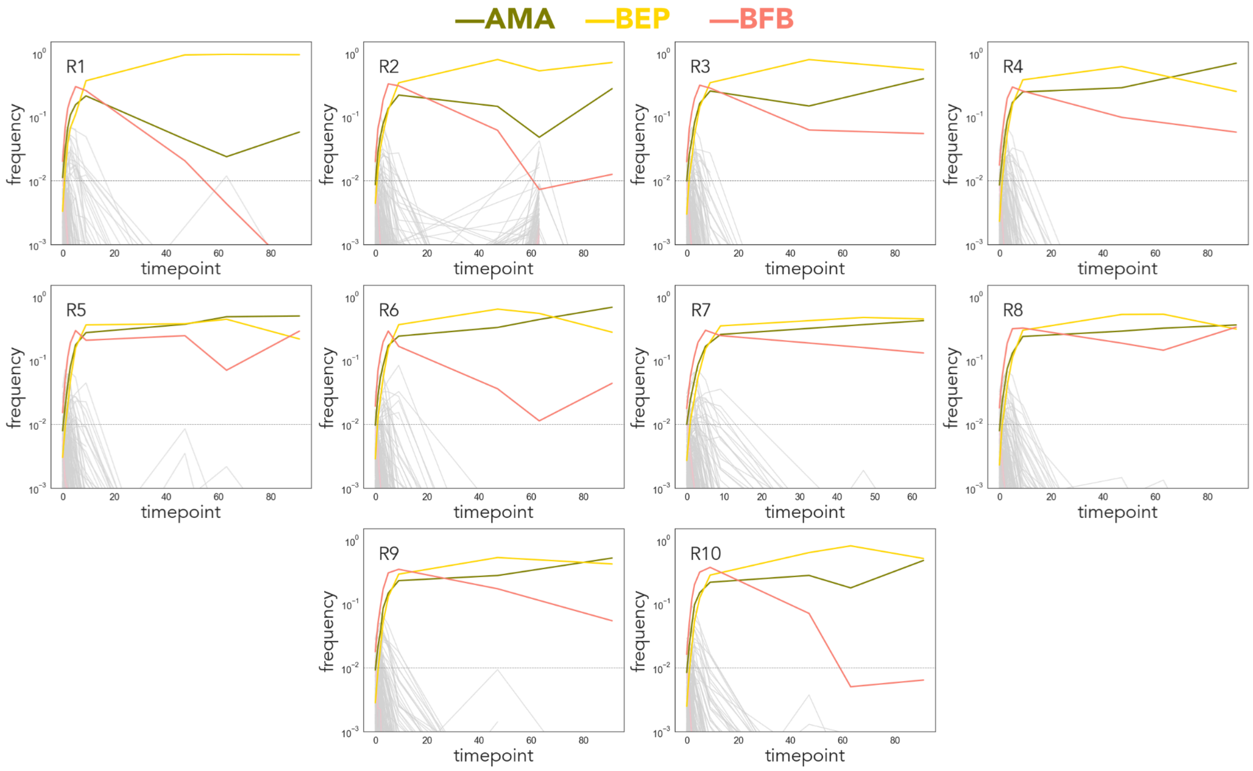

Fig. S5. Trajectories of the strains in the M3-5GLU environment with the finalist strains highlighted.

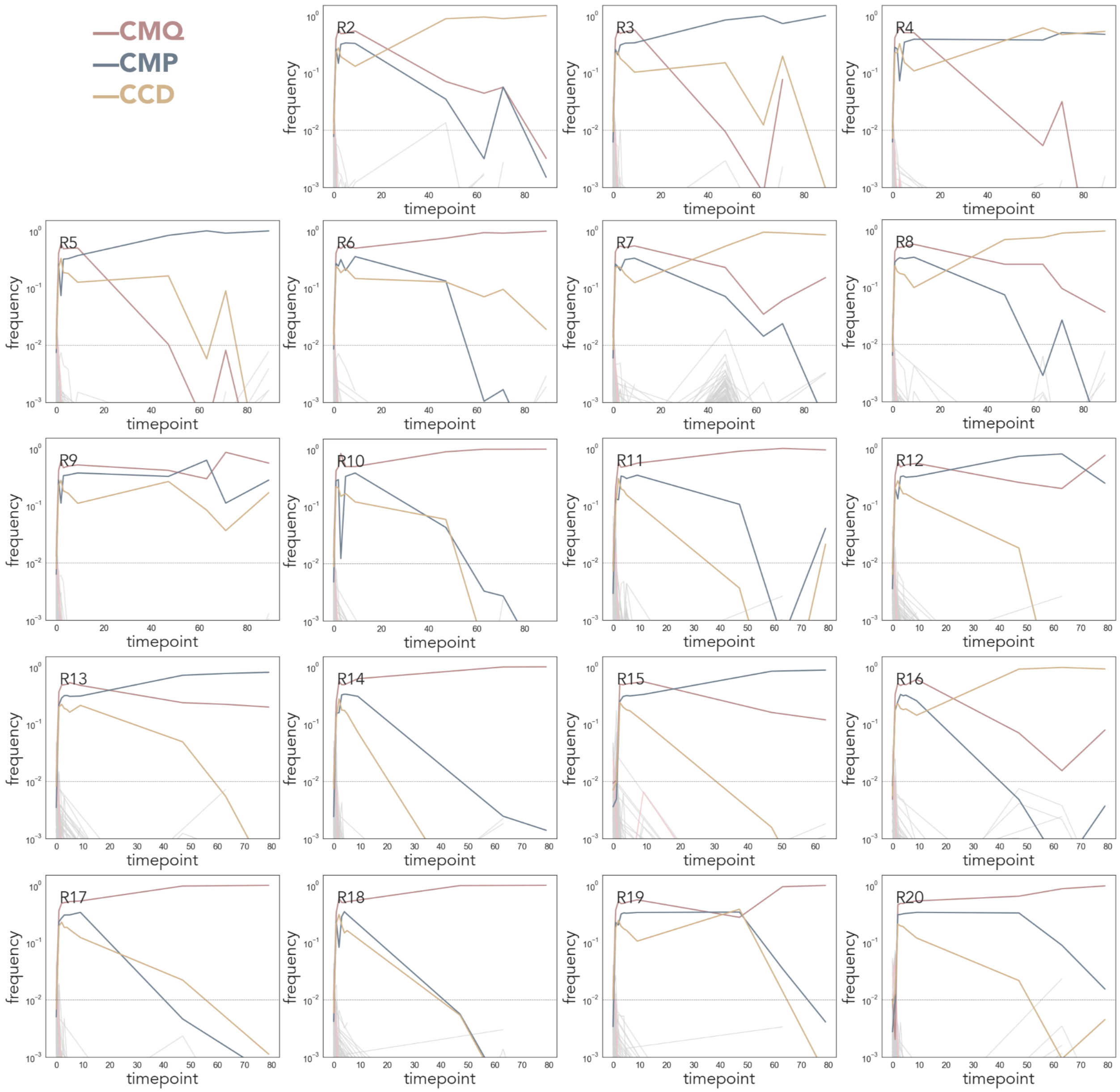

Fig. S6. Trajectories of the strains in the SC-HU environment with the finalist strains highlighted.

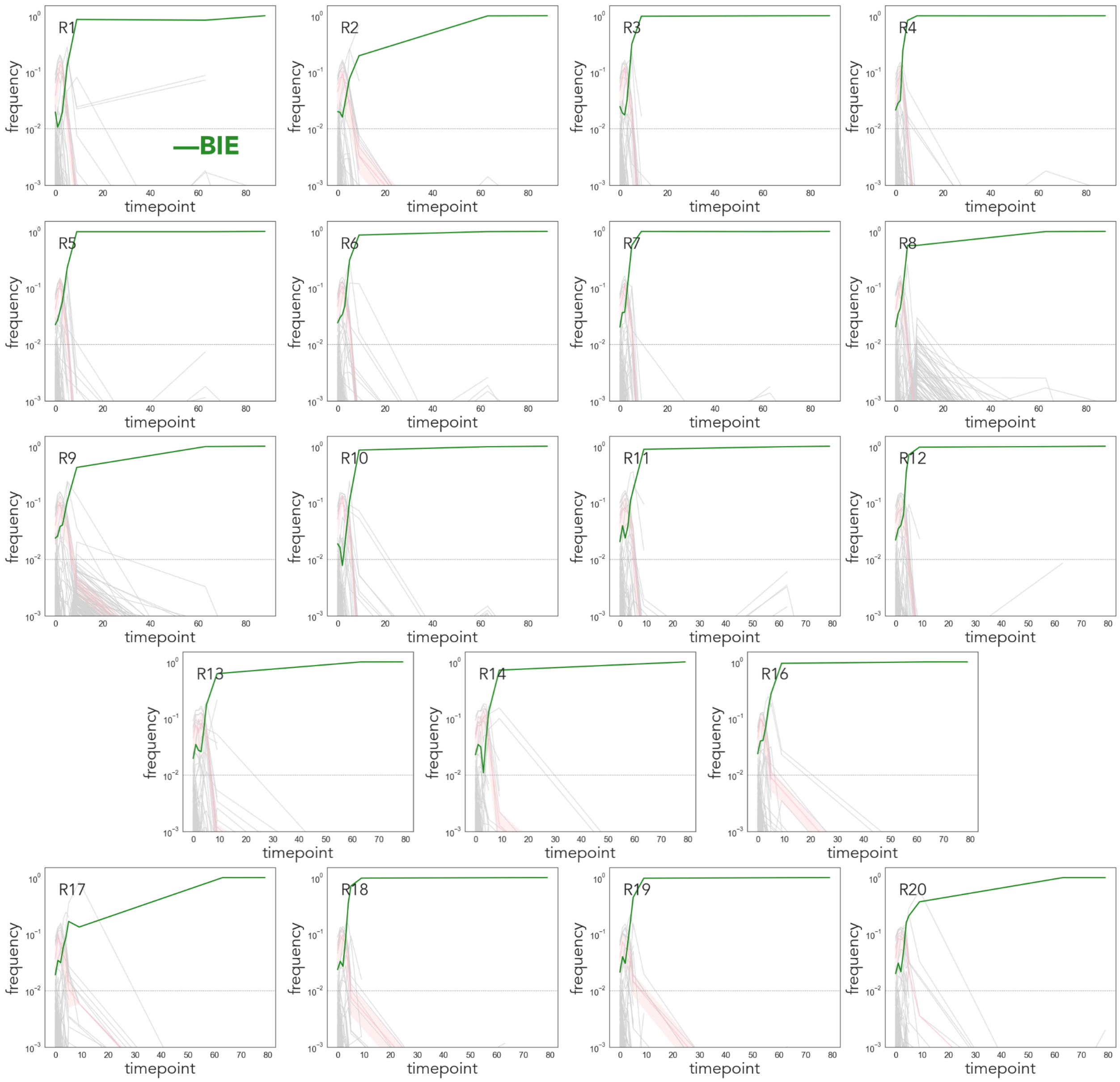

Fig. S7. Trajectories of the strains in the SC-SDS environment with the finalist strains highlighted.

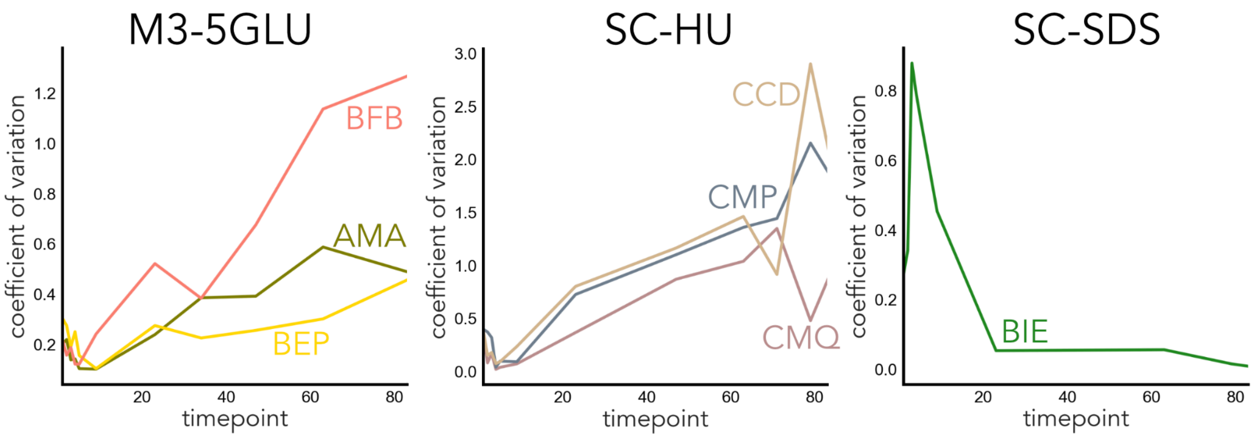

Fig. S8. Changes in the coefficients of variation for the finalist strains over time.

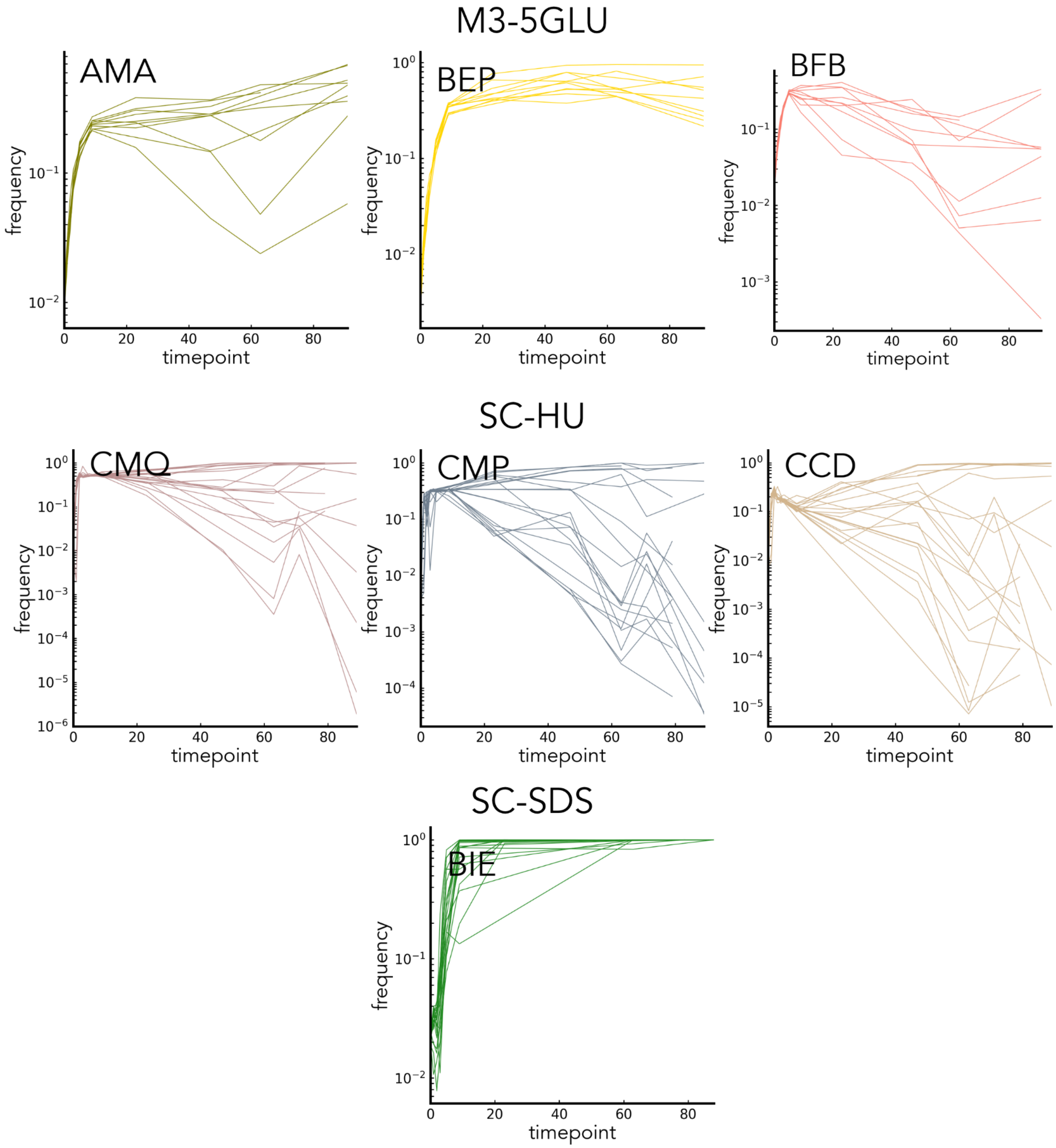

Fig. S9. Trajectories of the finalist strains across all experimental replicates.

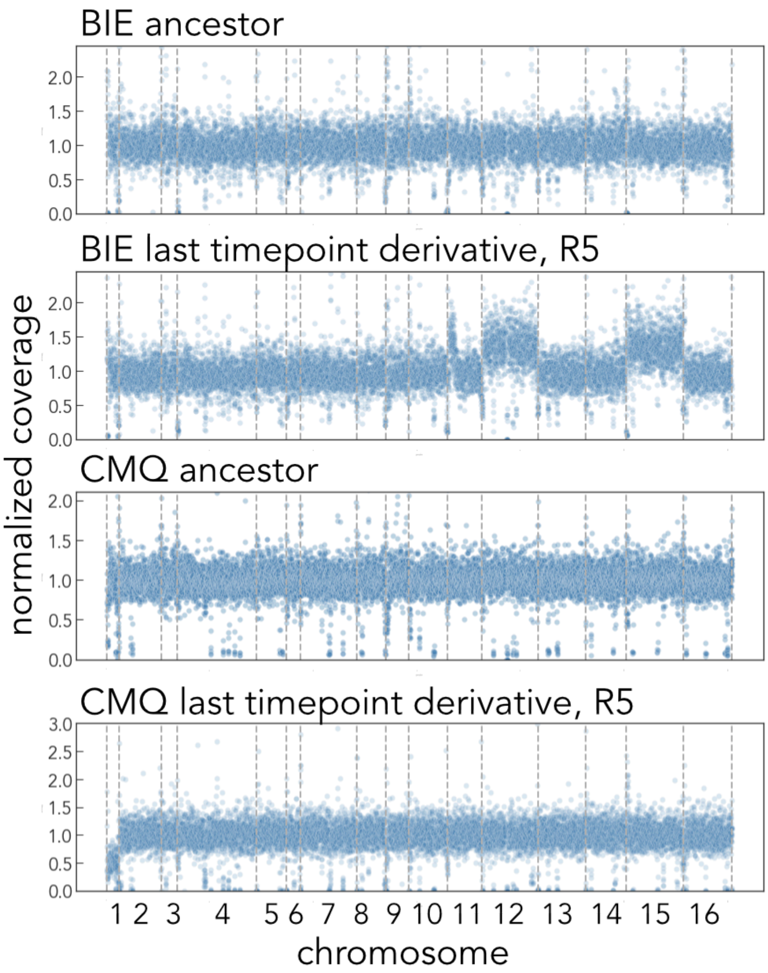

Fig. S10. Examples of normalized average coverage along 1 kb windows of the genome. Aneuploidies and segmental amplifications are detectable via excessive or depleted coverage across a region of the genome. Dashed vertical lines represent boundaries between chromosomes.
